# An exploratory study of the MABC-2 Checklist in Hungarian preschool children: agreement with examiner-based psychomotor classification

**DOI:** 10.3389/fpsyg.2026.1880230

**Published:** 2026-10-26

**Authors:** Kinga Révész-Kiszela, Gabriella Trzaskoma-Bicsérdy, László Révész

**Affiliations:** 1 Institute of Special Education, Eszterházy Károly Catholic University, Eger, Hungary; 2 Sport and Health Science Research Group, Eszterházy Károly Catholic University, Eger, Hungary; 3 Institute of Sports Science, Eszterházy Károly Catholic University, Eger, Hungary

**Keywords:** exploratory agreement, MABC-2 Checklist, motor assessment, preschool children, psychomotor functioning, school readiness

## Abstract

**Background:**

Early motor and broader developmental functioning are relevant to successful school entry, but cross-sectional screening procedures cannot by themselves determine overall school readiness or predict later academic outcomes.

**Objective:**

This exploratory study examined selected psychomotor indicators in a Hungarian preschool sample and compared teacher-based MABC-2 Checklist classifications with a study-specific examiner-based psychomotor classification, and examined the pattern of disagreement between the two procedures.

**Methods:**

Seventy-three children aged 5–7 years (mean age 6.46 ± 0.44 years) from three urban kindergarten classes in Pest County, Hungary, participated in the study. The assessment included anthropometric measurements, primitive-reflex observations, and gross-motor task performance, which were combined into a three-category examiner-based classification using an explicit decision framework. Preschool teachers completed the MABC-2 Checklist. Agreement between the two ordered three-category classifications was examined using cross-tabulation, percentage agreement, and linearly weighted Cohen’s kappa with a bootstrap 95% confidence interval; sensitivity and specificity of the Checklist relative to the examiner-based classification were calculated as exploratory indices of convergence.

**Results:**

Mean height was within the reference range used in the study, whereas mean body weight was descriptively somewhat higher and should be interpreted cautiously. Based on the teacher-based MABC-2 Checklist, 43.8% of children were categorized as at risk of motor difficulty or as showing motor difficulty; the corresponding proportion for the examiner-based classification was 50.7%. The two classification approaches were concordant in 71.2% of cases; linearly weighted Cohen’s kappa was 0.503 (95% CI 0.315–0.677), indicating moderate agreement. Agreement was lowest in the at-risk category (63.6%), and 9 of the 21 discordant cases differed by two categories, almost always with teachers rating as unproblematic children whom the examiner classified as showing motor difficulty. Relative to the examiner-based classification, the sensitivity of the Checklist was 70.3% and its specificity 83.3%.

**Conclusion:**

In this exploratory Hungarian preschool sample, the MABC-2 Checklist showed partial agreement with an examiner-based psychomotor classification. Because the examiner-based procedure was study-specific and non-standardized, and because the developmental significance of primitive-reflex indicators remains contested, the agreement estimates describe convergence between two rater-dependent procedures rather than validity. These findings do not constitute formal validation of the checklist and should not be interpreted as evidence of overall school readiness or prediction of later academic outcomes. Larger studies using formally adapted instruments, reliability analyses, standardized reference procedures, and longitudinal follow-up are needed before broader standalone use can be recommended.

## 1 Introduction

School readiness is a multidimensional construct that includes cognitive, social-emotional, and physical or psychomotor domains. Early childhood abilities at school entry are associated with later educational adaptation and achievement, and motor skills form one relevant component of this broader developmental profile (Kamphorst et al., 2021; Pagani et al., 2010; Snow, 2006).

In practice, concerns about school entry may arise even among children without identified special educational needs when individual developmental domains do not align with age-related expectations. School maturity therefore remains an important concept in educational practice, although its definition and assessment are still debated (Calsamiglia and Loviglio, 2020; McCartan et al., 2023; Snow, 2006).

Fundamental movement skills (FMS) contribute to children’s physical, cognitive, and social development. Within early childhood education, the quality of movement opportunities and the professional preparation of educators may influence motor development. Honrubia Montesinos et al. (2023) reported that professional development in physical education can strengthen teachers’ capacity to promote FMS in young children.

Motor competence, including gross- and fine-motor skills, has also been associated with early cognitive and academic outcomes. Rodríguez-Guerrero et al. (2023) reported positive relationships between motor skill proficiency and language and mathematical development in young children, supporting the view that motor development should be considered within a broader developmental framework.

Motor development is multifactorial and may be influenced by educational context, opportunities for structured and spontaneous physical activity, and individual developmental variation (Honrubia Montesinos et al., 2023; Rodríguez-Guerrero et al., 2023). These factors should be considered when interpreting screening results in preschool populations.

Together, these findings support the inclusion of motor functioning as one component of a broader developmental assessment while also indicating that motor measures alone cannot determine overall school readiness.

Difficulties observed at school entry may involve multiple domains and should therefore not be attributed to a single developmental indicator. Studies of school-readiness profiles suggest that motor skills can contribute information alongside cognitive and non-cognitive measures (Kamphorst et al., 2021; Pagani and Messier, 2012; Pagani et al., 2010).

Accordingly, motor competence may provide useful supplementary information within school-entry assessment, but it should not be treated as a standalone indicator of overall school readiness or later academic performance (Kamphorst et al., 2021; Pagani and Messier, 2012; Snow, 2006).

Within such frameworks, motor competence is thought to contribute to school readiness through several pathways. Fine-motor and visuomotor skills underpin early writing, drawing, and self-care tasks in the classroom; gross-motor and balance skills enable participation in play and physical activity, through which social skills, attention, and self-regulation are practiced; and shared neural and cognitive resources, including executive functions, have been proposed to link motor and cognitive development (Kamphorst et al., 2021; Pagani and Messier, 2012; Rodríguez-Guerrero et al., 2023). Profile-based analyses suggest that children’s motor skills at school entry are relevant to both cognitive and non-cognitive first-grade outcomes (Kamphorst et al., 2021). Motor competence is therefore best viewed as one interacting dimension of a multidimensional readiness profile rather than as an isolated prerequisite, and motor screening is most informative when embedded in a broader developmental assessment (Pagani and Messier, 2012; Snow, 2006).

One instrument designed to support the identification of children with possible motor coordination difficulties is the Movement Assessment Battery for Children-2 Checklist (MABC-2 Checklist), an observational motor screening checklist intended for completion by adults who know the child in everyday settings (Henderson et al., 2007; Schoemaker et al., 2012). The instrument has not yet undergone a formal Hungarian-language cultural adaptation process.

School readiness and school maturity are related but not identical concepts. In this paper, school readiness is used as the broader term encompassing cognitive, social-emotional, and physical or psychomotor preparedness for school entry, whereas school maturity refers more specifically to the somatic and psychomotor aspects emphasized in Hungarian educational practice (McCartan et al., 2023; Snow, 2006). This terminology is applied consistently throughout the manuscript.

This distinction is important because readiness for learning cannot be inferred from motor performance alone. Motor observations may contribute to a broader assessment, but conclusions about school entry require consideration of multiple developmental domains.

The MABC-2 manual reports evidence concerning the checklist’s ability to identify motor difficulties in clinical samples and describes the traffic-light classification system used to indicate typical performance, risk, and more pronounced motor difficulty (Henderson et al., 2007). These findings support the checklist’s role as a screening instrument rather than as a standalone diagnostic measure.

Schoemaker et al. (2012) examined the psychometric properties of the motor component of the MABC-2 Checklist in 203 children aged 5–8 years. They reported good internal consistency and evidence that checklist items differentiated children with and without motor impairments, while also showing expected age-related developmental patterns.

Capistrano et al. (2015) examined agreement among different approaches to identifying motor difficulties and reported differences according to the type of evaluator. Earlier work by Junaid et al. (2000) likewise emphasized that the accuracy of observation-based screening may depend on the rater’s experience and familiarity with children’s motor behavior.

Not all studies have found strong agreement between the MABC-2 Checklist and other motor assessments. de Milander et al. (2019), studying 281 Grade 1 learners and their parents, reported low agreement between parent-completed checklist classifications and motor-test results. Such findings indicate that agreement can vary according to rater type, context, and comparison procedure and reinforce the need for cautious interpretation of checklist classifications.

In Hungarian developmental–pedagogical practice, assessments of school maturity frequently include observations of residual primitive-reflex activity alongside gross-motor performance. Primitive reflexes are brainstem-mediated motor responses that are normally inhibited during the first year of life. Their persistence beyond infancy has been proposed, particularly within the framework of the Institute for Neuro-Physiological Psychology (INPP), as a marker of neuromotor immaturity that may be associated with difficulties in balance, coordination, attention, and learning (Blythe, 2005; Goddard Blythe, 2005; Melillo et al., 2022).

This interpretation is contested, and the evidence base should be read with caution. Residual reflex activity is common in children without any developmental diagnosis: Gieysztor et al. (2018) observed residual-level reflex activity in 65% of 35 healthy 4–6-year-olds, Pecuch et al. (2020) likewise documented active primitive reflexes in a sample of 44 healthy preschoolers and summarized reports that such activity is present in the majority of healthy preschool children, and Hickey and Feldhacker (2022) found at least one retained reflex in each of 27 typically developing preschool children. Although these and other studies report associations between stronger reflex activity and poorer balance, coordination, or attention, the available evidence is almost entirely cross-sectional, based on small samples, and partly produced by groups that also develop reflex-based intervention programmes; the construct validity, diagnostic utility, and predictive value of retained reflexes for motor competence and school outcomes have not been established. Reviews of perceptual-motor and reflex-based educational programmes have found little evidence of educational benefit (Hyatt et al., 2009), and the American Occupational Therapy Association has advised against reflex-integration programmes without a clear link to functional outcomes, noting that the presence of retained reflexes does not necessarily equate to functional impairment (American Occupational Therapy Association, 2021). In the present study, reflex observations were therefore treated as one descriptive component of a broader psychomotor profile and not as a diagnostic indicator of developmental immaturity or educational risk.

The present study was designed as an exploratory investigation of selected anthropometric and psychomotor characteristics in a Hungarian preschool sample and of the agreement between teacher-based MABC-2 Checklist classifications and an examiner-based psychomotor classification. It was not designed to establish overall school readiness, predictive validity, or formal psychometric validation of the MABC-2 Checklist.

Accordingly, the objectives were:

1. To describe selected anthropometric and psychomotor characteristics of the sample;
2. To identify children who may show psychomotor deviations within this sample;
3. To examine the descriptive agreement between teacher-based MABC-2 Checklist categories and an examiner-based psychomotor classification;
4. To discuss the practical and methodological implications of using such procedures in Hungarian preschool settings.

The exploratory expectations of the study were that:

1. The sample would, on average, fall within broadly expected anthropometric ranges, although individual variation might be present;
2. Some children attending mainstream kindergarten classes would nevertheless show psychomotor deviations on the examiner-based assessment;
3. The MABC-2 Checklist and examiner-based psychomotor classification would show partial, but not complete, agreement.

## 2 Materials and methods

### 2.1 Participants and sampling

We used non-probability expert sampling in three urban kindergarten classes in Pest County, Hungary (*n* = 73; 43 boys and 30 girls; mean age 6.46 ± 0.44 years). Inclusion criteria were attendance in the final kindergarten year, parental consent, and availability of teacher-completed checklist data. Children with a documented neurological or developmental disorder and children with missing data on key measures were excluded. Because the absence of a documented diagnosis does not by itself establish typical development, the sample is described throughout as children attending mainstream kindergarten without documented neurological or developmental diagnoses.

Two kindergarten teachers were assigned to each of the three participating classes (*n* = 6 in total). Rather than completing the Checklist independently, the two teachers of each class jointly reviewed the items for every child in their group and agreed on a single consensus rating for each item. This procedure was chosen deliberately: both teachers observed the children daily in the same educational setting, and a consensus-based rating was considered to reflect their shared everyday observational knowledge of the child more faithfully than two separate ratings obtained from partially overlapping observational contexts. Accordingly, one consensus-based Checklist classification was obtained per child, and this classification was used in the agreement analysis.

The six participating teachers were all qualified preschool teachers holding a Bachelor’s degree (BA) in Early Childhood Education, with 5–20 years of professional experience (median: 12 years). All teachers had at least 3 years of prior experience with motor assessment or developmental screening instruments, specifically the Meeting Street School Screening Test (MSSST) procedure. However, none had previous experience using the MABC-2 Checklist. Before data collection, the teachers received a detailed briefing from the principal investigator regarding the study’s purpose, duration, and procedures, as well as specific instructions on completing the MABC-2 Checklist, including the interpretation of response categories and the appropriate use of the “not observed” option. The completed Checklists were evaluated by the principal investigator. No formal rater training or calibration exercise against a reference standard was conducted, which is acknowledged as a limitation of the study.

Because a single consensus rating was produced per child rather than two independent ratings, inter-rater reliability between the two teachers of a class cannot be computed from the present data. We acknowledge this as a consequence of the chosen procedure and note it as a limitation: while consensus rating increases the ecological grounding of the classification, it does not allow the degree of independent agreement between individual educators to be quantified. Future studies should obtain independent parallel ratings in order to estimate inter-rater reliability directly.

### 2.2 Motor assessment and scoring

Rationale for the examiner-based classification. To our knowledge, no standardized motor assessment with Hungarian normative data for this age group was available at the time of the study, and the MABC-2 Test has not been standardized for Hungarian children. The examiner-based procedure therefore combined two components that are widely used in Hungarian developmental–pedagogical practice: an assessment of residual primitive-reflex activity according to the INPP protocol (Goddard Blythe, 2005), and a set of gross-motor tasks targeting balance, locomotor control, and spatial orientation. The two components were conceptualized as different levels of the same psychomotor profile: residual reflex activity is assumed to constrain postural control and bilateral coordination, whereas the gross-motor tasks capture the functional expression of these capacities. Associations between stronger residual reflex activity and poorer motor proficiency have been reported in healthy preschool children (Gieysztor et al., 2018), but these associations are observational, and the clinical and educational significance of residual reflex activity remains debated (see sections “1 Introduction” and “4 Discussion”). The composite classification was therefore developed as a pragmatic comparator for the present exploratory study. It is not a validated reference standard, and the agreement estimates reported below quantify convergence between two rater-dependent procedures rather than the validity of either.

Primitive reflex activity was assessed using the standardized protocol of the Institute for Neuro-Physiological Psychology (INPP), as described by Goddard Blythe (2005). The asymmetrical tonic neck reflex (ATNR), symmetrical tonic neck reflex (STNR), tonic labyrinthine reflex (TLR), Galant reflex, Landau reflex, and Moro reflex were elicited and scored following the test positions, elicitation procedures, and grading criteria specified in that protocol. Reflex activity was graded on the protocol’s 0–4 scale, where 0 denotes a fully integrated (absent) response and 1, 2, 3, and 4 denote approximately 25%, 50%, 75%, and 100% retention of the response, respectively.

The reflex assessments were administered and scored by examiners holding the internationally recognized INPP practitioner qualification, which comprises formal training in the elicitation, grading, and interpretation of primitive and postural reflexes according to the INPP protocol. This qualification is specific to the procedure used here and extends beyond general professional experience in movement development or preschool psychomotor assessment. All reflex assessments were conducted according to the same protocol and grading criteria across the three participating classes.

Additional gross-motor tasks were used to observe locomotor control, spatial orientation, and balance-related performance. Multiple attempts were allowed when necessary. The tasks were:

1. Forward heel-to-toe walking in a straight line (10 m)
2. Backward heel-to-toe walking in a straight line (10 m)
3. Walking forward in a straight line on the outer sole (10 m)
4. Walking backward in a straight line on the outer sole (10 m)
5. Crawl forward (10 m)
6. Climb forward (10 m)
7. Walking with knee touch (cross touch) (10 m)

For the purposes of this exploratory study, gross-motor task performance was scored on a 0–4 ordinal scale. A score of 0 indicated performance consistent with the expected task execution, 1 indicated a minor deviation, 2 indicated clearly inadequate execution, 3 indicated markedly incorrect execution, and 4 indicated inability to complete the task. All examiner-based assessments were conducted by the first author, who has professional experience in movement development and preschool psychomotor assessment. The scoring system was study-specific and should not be interpreted as a standardized diagnostic instrument.

The primitive-reflex and gross-motor scores were interpreted according to the meaning of each underlying scale rather than as psychometrically equivalent numerical values. In the INPP protocol, scores of 1 and 2 indicate residual reflex activity of increasing magnitude, but do not, by themselves, indicate that developmental intervention is required. Scores of 3 and 4 indicate substantial reflex retention, for which developmental intervention is considered strongly indicated within the INPP framework. In contrast, a score of 2 on a gross-motor task denotes clearly inadequate task execution, while scores of 3 and 4 indicate more severe performance difficulty or inability to complete the task.

Accordingly, the use of the same numerical values across the two assessment components should not be interpreted as implying measurement equivalence. The common 0–4 format was used as a practical severity framework, but reflex and gross-motor scores retained their component-specific meanings. For descriptive reporting, scores of 2 or above were presented to identify any observable deviation from optimal performance. However, in the examiner-based classification, a reflex score of 2 was treated as residual reflex activity rather than as equivalent to a gross-motor score of 2, and reflex scores of 3 or 4 were considered the clinically more relevant levels of retention. This distinction is made explicit here to prevent the descriptive threshold from being interpreted as a validated common diagnostic cut-off.

The final examiner-based classification integrated the pattern and severity of findings across the two assessment components. Although no arithmetic algorithm was applied at the time of assessment, the examiners’ judgment followed a consistent set of working criteria, which are reported here in operational form (Table 1) to make the procedure as transparent and replicable as possible. Children with no meaningful gross-motor deviation and no substantial reflex retention–operationally, all six reflexes scored 0 or 1, no gross-motor task scored 3 or 4, and at most [one] task scored 2–were classified as having no motor difficulty. Children showing limited gross-motor deviation and/or residual reflex activity without substantial retention–at least one reflex scored 2 but none scored 3 or 4, and/or [two] or more tasks scored 2 or one task scored 3 or 4–were classified as being at risk of motor difficulty. Children showing marked gross-motor impairment and/or substantial reflex retention–at least one reflex scored 3 or 4, and/or [two] or more tasks scored 3 or 4–were classified as having motor difficulty. Where the two components pointed to different categories, the more severe category was assigned. Classification was based on the overall pattern of findings rather than on the arithmetic summation of psychometrically equivalent scores. Applying the tabulated criteria retrospectively to the recorded scores reproduced the assigned category for 50 of the 73 children (68, 49%); in the remaining 23 cases the examiner assigned a more/less severe category on the basis of the overall pattern.

**TABLE 1:** Decision framework used for the examiner-based psychomotor classification.

Category	Primitive-reflex component (INPP score, 0–4)	Gross-motor component (task score, 0–4)	Rule for combining the components
No motor difficulty	All six reflexes scored 0 or 1	No task scored 3 or 4; at most one task scored 2	Both conditions required
At risk of motor difficulty	At least one reflex scored 2; none scored 3 or 4	Two or more tasks scored 2, or one task scored 3 or 4	Either condition, provided that the criteria for motor difficulty are not met
Motor difficulty	At least one reflex scored 3 or 4	Two or more tasks scored 3 or 4	Either condition

Scores retain their component-specific meanings (section “2.2 Motor assessment and scoring”): a reflex score of 2 denotes residual activity and scores of 3–4 denote substantial retention, whereas a gross-motor score of 2 denotes clearly inadequate execution and scores of 3–4 denote markedly incorrect execution or inability to complete the task. Where the two components indicated different categories, the more severe category was assigned. The thresholds are pragmatic working criteria established for this exploratory study and are not validated cut-offs.

No fixed arithmetic algorithm or formally validated cut-off was used to combine the two components, and the thresholds in Table 1 were chosen pragmatically rather than derived from normative data. The final category was assigned by the qualified INPP examiners on the basis of the overall pattern and severity of gross-motor and reflex findings within this framework. We state this explicitly and acknowledge that the reliance on examiner judgment, the absence of a validated reference standard, and the study-specific thresholds limit the reproducibility of the classification and the interpretability of the agreement estimates. The kappa coefficient reported below therefore quantifies convergence between two rater-dependent procedures; its upper bound is set by the reliability of the less reliable procedure, which for the examiner-based classification is unknown.

### 2.3 MABC-2 Checklist

The MABC-2 Checklist is an observational motor assessment checklist developed by Henderson et al. (2007) as part of the Movement Assessment Battery for Children-2. It is intended to support the identification of children whose everyday motor behavior may indicate coordination difficulties.

The checklist focuses on observable motor behavior in everyday settings such as the classroom and playground, including activities such as fastening clothing, using scissors, and throwing or catching a ball.

The MABC-2 Checklist contains 30 motor items for children aged 5–12 years and is designed for completion by adults who are familiar with the child’s everyday motor behavior (Henderson et al., 2007; Schoemaker et al., 2012).

Items are rated on a 0–3 scale reflecting the quality of task performance; a separate “not observed” response is available when the rater has not had an opportunity to observe the activity. Checklist scores are interpreted according to the age-related scoring procedures and traffic-light categories described in the MABC-2 manual (Henderson et al., 2007).

The traffic-light interpretation distinguishes children whose observed motor behavior falls within the expected range, children who may be at risk of motor difficulty, and children whose scores indicate more pronounced motor difficulty; in the MABC-2 system these bands are anchored to the 5th and 15th percentiles of the standardization sample (Henderson et al., 2007). In the present study, these three ordered categories were used for comparison with the examiner-based psychomotor classification.

The Hungarian version of the Checklist items used in this study was prepared through forward translation from the original English-language manual by the second author in collaboration with a bilingual specialist in special education. The Hungarian version was subsequently back-translated into English by the first author, and the back-translated version was reviewed by a bilingual subject-matter expert. No psychometric evaluation of the translated version (item equivalence, internal consistency, test–retest or inter-rater reliability) was undertaken, and the original age-related scoring procedures and cut-offs of the manual (Henderson et al., 2007) were applied. Several items refer to activities that are rarely observable in Hungarian kindergarten routines (for example, carrying a tray among moving peers); such items may have elicited “not observed” responses more often, which can lower total scores and affect category assignment. Linguistic equivalence, cultural appropriateness, and the interpretability of the scores for Hungarian preschool children therefore remain to be established, and the use of the Checklist without a formal Hungarian-language cultural adaptation is treated as a limitation of the study.

The checklist covers motor behavior in the following broad contexts:

1. Movement in a constant and/or predictable environment (tying buttons, bypassing colleagues standing in one place with a tray, avoiding fixed devices in the gym without collision, etc.),
2. Movement in a dynamic and/or unpredictable environment (dodging people in motion with a tray in hand, moving to the beat of music, continuously driving the ball, etc.),
3. Non-motor factors that may influence observed movement performance, such as attention, confidence, persistence, or anxiety (Henderson et al., 2007).

### 2.4 Statistical analysis

The analyses were descriptive and exploratory. Frequencies and percentages were used to summarize anthropometric and psychomotor findings. Agreement between the teacher-based MABC-2 Checklist categories and the examiner-based psychomotor categories was first described using a 3 × 3 cross-classification table and overall and category-specific percentage agreement. Because the categories are ordered, agreement beyond chance was additionally quantified using linearly weighted Cohen’s kappa. A 95% confidence interval for weighted kappa was estimated by nonparametric bootstrap resampling of the observed paired classifications (100,000 resamples). Weighted kappa was interpreted with reference to the conventional benchmarks of Landis and Koch (1977), with the caveat that such benchmarks are descriptive conventions rather than absolute standards. The analyses were interpreted as evidence of agreement between two rater-dependent classification procedures, not as formal psychometric validation against a diagnostic gold standard.

In exploratory supplementary analyses, the pattern of disagreement was examined by distinguishing one-category from two-category discrepancies and by inspecting their direction. The three categories were also dichotomized (no motor difficulty versus at risk of motor difficulty or motor difficulty), and the sensitivity, specificity, and positive and negative predictive values of the Checklist were calculated with the examiner-based classification as the comparator; because this comparator is not a validated reference standard, these indices describe convergence between the two procedures rather than diagnostic accuracy.

For the agreement analysis, both procedures were represented by the same three ordered categories: 1 = no motor difficulty, 2 = at risk of motor difficulty, and 3 = motor difficulty. The examiner-based category was derived from the study-specific composite of gross-motor task and primitive-reflex scores described above.

### 2.5 Ethics approval and informed consent

The children participated in the research during regular kindergarten sessions. The head teachers of the participating institutions and the children’s parents were contacted to obtain permission for data collection, and the researchers were present throughout the assessment sessions. Parents were informed about the assessment protocol and the intended use of the findings and gave written informed consent for their children’s participation; the children’s verbal assent was also obtained before each assessment. The study was conducted in accordance with the Declaration of Helsinki. Participation was voluntary, and all data were handled anonymously. All procedures were approved by the Ethics Committee of Eszterházy Károly Catholic University (RK/200/2025).

## 3 Results

### 3.1 Anthropometric characteristics of the entire sample

The mean height of the 73 children was 123.0 cm (SD = 5.5), and mean body weight was 23.8 kg (SD = 4.5). Mean height fell within the age-related reference range used in the study. Mean body weight was descriptively somewhat higher than the selected reference values, but no inferential conclusion about overweight, obesity risk, physical activity, or lifestyle can be drawn from these data alone.

These anthropometric findings are therefore presented descriptively and should not be interpreted as indicators of overall school readiness or as evidence of a particular lifestyle pattern.

### 3.2 Movement status assessment of the entire sample

Table 2 presents the percentage of children who received a score of ≥2 on each examiner-rated gross-motor task or primitive-reflex observation. This threshold represents the study-specific exploratory scoring rule and is not a diagnostic cut-off.

**TABLE 2:** The proportion of children showing difficulty in each movement test.

The examined motor tests	Score 2 (%)	Score 3 (%)	Score 4 (%)	Score ≥2 (%)
Forward heel-to-toe walking	23.3	11	11	45.3
Backward heel-to-toe walking	30.1	13.7	8.2	52
Walking on the outer edge of the foot forward	31.5	20.5	6.8	58.8
Walking on the outer edge of the foot backward	26	30.1	16.4	72.5
Crawling	15.1	21.9	23.3	60.3
Climbing	19.2	4.1	1.4	24.7
Walking with knee touch	26	6.8	4.1	36.9
ATNR reflex	17.8	19.2	8.2	45.2
STNR reflex	19.2	6.8	2.7	28.7
TLR reflex	20.5	6.8	5.5	32.8
Galant reflex	8.2	8.2	1.4	17.8
Landau reflex	17.8	2.7	0	20.5
Moro reflex	27.4	8.2	0	35.6

The final column reports the percentage of children with scores ≥2; scores 0 and 1 are not displayed.

On the gross-motor tasks, 45.3% of children received a score of ≥2 for forward heel-to-toe walking and 52.0% for backward heel-to-toe walking. The corresponding proportions were 58.8% for forward walking on the outer edge of the foot, 72.5% for the backward version, 60.3% for crawling, 24.7% for climbing, and 36.9% for walking with knee touch. These percentages indicate the frequency of deviations according to the study-specific scoring procedure and should not be interpreted as prevalence estimates of developmental coordination disorder.

Among the primitive-reflex observations, 45.2% of children received a score of ≥2 for the ATNR, 35.6% for the Moro reflex, 32.8% for the TLR, and 28.7% for the STNR. As with the motor tasks, these findings describe performance within the present assessment procedure and do not establish neuromotor immaturity or school unreadiness.

### 3.3 Agreement between MABC-2 Checklist and examiner-based psychomotor classifications

Based on the teacher-based MABC-2 Checklist, 32 of 73 children (43.8%) were categorized as at risk of motor difficulty (*n* = 22) or as showing motor difficulty (*n* = 10). Based on the examiner-based psychomotor classification, 37 of 73 children (50.7%) fell into these two categories (at risk, *n* = 18; motor difficulty, *n* = 19).

Table 3 cross-classifies the teacher-based MABC-2 Checklist categories (rows) and the examiner-based psychomotor categories (columns). The two procedures assigned the same category to 52 of 73 children, corresponding to an overall raw agreement of 71.2%. Category-specific agreement was 73.2% among children classified by the MABC-2 Checklist as having no motor difficulty, 63.6% in the at-risk category, and 80.0% in the motor-difficulty category. Viewed from the examiner-based categories, agreement was 83.3% (30/36) for no motor difficulty, 77.8% (14/18) for at risk, and 42.1% (8/19) for motor difficulty.

**TABLE 3:** Cross-classification of teacher-based MABC-2 Checklist categories (rows) and examiner-based psychomotor categories (columns).

Teacher-based MABC-2 Checklist category (rows)/examiner-based psychomotor category (columns)	No motor difficulty	At risk of motor difficulty	Motor difficulty	Total
No motor difficulty	30	3	8	41
At risk of motor difficulty	5	14	3	22
Motor difficulty	1	1	8	10
Total (examiner-based)	36	18	19	73
No motor difficulty (%)	73.2	7.3	19.5	100
At risk of motor difficulty (%)	22.7	63.6	13.6	100
Motor difficulty (%)	10	10	80	100

Rows show the teacher-based MABC-2 Checklist category and columns the examiner-based psychomotor category. Values in the lower part of the table are row percentages. Concordant cases (diagonal) total 52 of 73 (71.2%).

When the ordinal structure of the three categories and agreement expected by chance were taken into account, the linearly weighted Cohen’s kappa was 0.503 (95% CI 0.315–0.677). According to conventional benchmarks (Landis and Koch, 1977), this value indicates moderate agreement, although the lower confidence limit falls within the range usually described as fair. Thus, the two classification procedures showed partial agreement beyond chance, but the confidence interval also indicates uncertainty associated with the modest sample size.

The lowest category-specific agreement occurred in the at-risk group (63.6%). This intermediate category may be particularly sensitive to differences in observational context, scoring thresholds, and evaluator judgment. Accordingly, the results support cautious use of the checklist as one component of a broader assessment process rather than as a standalone classification tool.

Of the 21 discordant classifications, 12 differed by one category and 9 by two categories. Eight of the nine two-category discrepancies involved children rated by their teachers as having no motor difficulty but classified by the examiner as showing motor difficulty; the reverse pattern occurred once. Discrepancies were thus not confined to borderline cases, and the examiner-based procedure tended to assign more severe categories than the teachers (Table 3). When the categories were dichotomized and the examiner-based classification was used as the comparator, the Checklist identified 26 of the 37 children in the two higher examiner-based categories (sensitivity 70.3%) and 30 of the 36 children without examiner-identified difficulty (specificity 83.3%); the positive and negative predictive values were 81.3% (26/32) and 73.2% (30/41), respectively. For the motor-difficulty category alone, sensitivity was 42.1% (8/19) and specificity 96.3% (52/54). These indices describe convergence between two non-standardized procedures and should not be read as estimates of diagnostic accuracy.

## 4 Discussion

This exploratory study compared teacher-based MABC-2 Checklist classifications with an examiner-based psychomotor classification in a small Hungarian preschool sample. The principal finding was partial agreement between the two procedures: raw agreement was 71.2%, and linearly weighted Cohen’s kappa was 0.503 (95% CI 0.315–0.677), corresponding to moderate agreement by conventional benchmarks (Landis and Koch, 1977). These results indicate agreement beyond chance but do not establish formal validation of the MABC-2 Checklist, because the examiner-based procedure was itself non-standardized and rater-dependent.

The findings are relevant to early educational assessment because motor observations can contribute information about children’s functioning that may not be captured by cognitive or social-emotional measures alone. Nevertheless, motor performance represents only one component of the broader construct of school readiness (Kamphorst et al., 2021; Pagani and Messier, 2012; Snow, 2006). Within readiness frameworks, motor competence is thought to act both directly–through the fine-motor and postural demands of classroom tasks–and indirectly, by enabling participation in play and physical activity through which attention, self-regulation, and social skills are practiced (Kamphorst et al., 2021; Pagani and Messier, 2012; Rodríguez-Guerrero et al., 2023). Motor screening is therefore most informative when its results are interpreted together with cognitive, language, and social-emotional indicators, and when it informs support and further observation rather than placement decisions. The present results therefore support a complementary role for motor screening rather than the use of motor findings as a substitute for multi-domain school-entry assessment.

The cross-sectional design is especially important when interpreting the educational implications of the findings. Although motor competence has been associated with school-readiness profiles and later academic outcomes in previous research (Kamphorst et al., 2021; Pagani et al., 2010; Rodríguez-Guerrero et al., 2023), the present study did not follow children after school entry. Consequently, it cannot demonstrate that the observed psychomotor patterns predict later learning difficulties, school achievement, or educational adaptation.

The MABC-2 Checklist was developed as an observational screening instrument within the broader MABC-2 framework (Henderson et al., 2007), and previous research has examined its reliability and validity in different populations and rater groups (Capistrano et al., 2015; Schoemaker et al., 2003, 2012). The present study extends this line of work only in an exploratory sense by describing agreement in a Hungarian preschool sample. Because a formal Hungarian cultural adaptation has not yet been established, the current findings should not be interpreted as validation for national use.

The anthropometric findings should also remain descriptive. Mean height was within the reference range used in the study, while mean body weight was somewhat higher than the selected reference values. Because physical activity, body composition, nutritional intake, and related lifestyle variables were not measured, the present data do not support causal interpretations concerning sedentary behavior, overweight, or obesity risk.

The examiner-based procedure categorized 50.7% of the sample, and the teacher-based Checklist 43.8%, as at risk of motor difficulty or as showing motor difficulty. Both proportions are considerably higher than prevalence estimates for developmental coordination disorder, which are usually in the region of 5%–6% of school-aged children (Blank et al., 2019), and this requires explanation rather than a population-level inference. Several factors are likely to have contributed. First, the thresholds of the examiner-based framework were liberal by design: a score of 2 on a gross-motor task denotes a clearly visible but not severe deviation, and a reflex score of 2 denotes residual activity that is common in children of this age (Gieysztor et al., 2018; Hickey and Feldhacker, 2022; Pecuch et al., 2020); because a child entered the at-risk category on the basis of either component, the combined procedure necessarily flags more children than either component alone. Second, several of the gross-motor tasks (for example, backward heel-to-toe walking or walking on the outer edge of the foot) are demanding balance tasks on which many 5–7-year-olds show imperfect execution, as Table 2 illustrates; task performance is also sensitive to attention, motivation, and familiarity with the testing situation. Third, the sample was small, non-random, and drawn from three classes in one county, so class- or setting-specific factors, such as opportunities for structured movement, cannot be excluded. Fourth, the traffic-light bands of the MABC-2 are anchored to the 5th and 15th percentiles of the standardization sample (Henderson et al., 2007), so the teachers’ 43.8% also indicates a liberal rating tendency in this sample, which may reflect the consensus rating procedure, the unadapted Hungarian wording of the items, or the teachers’ interpretation of the response categories. The proportions should therefore not be interpreted as evidence that a comparable share of Hungarian preschool children have a developmental disorder or are unready for school; rather, they indicate that both procedures, as applied here, functioned as broad screens with low thresholds.

The agreement analysis provides a more informative summary than raw percentage agreement alone. Although 71.2% of classifications were identical, weighted kappa accounted for both chance agreement and the ordered nature of the three categories and yielded a linearly weighted kappa of 0.503 (95% CI 0.315–0.677), which corresponds to moderate agreement by the conventional benchmarks of Landis and Koch (1977). This value is higher than the kappa coefficients reported when teacher-completed MABC-2 Checklists were compared with the MABC-2 Test in Dutch (κ = 0.28; 10) and Greek samples (κ = 0.28 overall and 0.44 for preschool teachers; 25), and when parent-completed Checklists were compared with the MABC-2 Test in South African Grade 1 learners (κ = 0.16; 13); the sensitivity (70.3%) and specificity (83.3%) observed here are in the same range as, or above, the values of approximately 41%–51% and 81%–88% reported for teachers in the Dutch and Greek studies (Dimitropoulou et al., 2019; Schoemaker et al., 2012). This should not be interpreted as superior performance of the Checklist in the Hungarian sample. A more plausible explanation is that both procedures in the present study were observation-based ratings of movement quality made by adults, whereas the MABC-2 Test yields a standardized performance score; shared method characteristics are likely to inflate agreement. Moreover, weighted kappa is bounded by the reliability of the examiner-based procedure, which is unknown. In practical terms, a kappa of about 0.5 means that roughly half of the agreement not expected by chance was achieved; the two procedures therefore provide overlapping but clearly non-interchangeable information. The lower agreement in the at-risk category (63.6%) is particularly relevant because borderline classifications are likely to be more sensitive to differences in observation and threshold application than clearly typical or clearly problematic performance. At the same time, 9 of the 21 discordant cases differed by two categories, almost always in the direction of teachers rating as unproblematic children whom the examiner classified as showing motor difficulty; discrepancies were thus not confined to borderline cases. Consistent with earlier reports that the Checklist is more specific than sensitive (Dimitropoulou et al., 2019; Junaid et al., 2000; Schoemaker et al., 2012), this suggests that in a screening context a negative Checklist result should not be taken to exclude motor difficulty identified by direct observation.

Neither procedure should be treated as an objective gold standard. The MABC-2 Checklist depends on teachers’ everyday observations, while the examiner-based psychomotor classification also involved professional judgment during task scoring and combined measures that are not psychometrically equivalent. Previous studies have likewise shown that rater type and expertise can affect observation-based identification of motor difficulties (Capistrano et al., 2015; Dimitropoulou et al., 2019; Junaid et al., 2000). The present findings therefore describe the relationship between two different observational perspectives rather than validation against a diagnostic reference test.

Primitive-reflex observations require especially cautious interpretation. In this study, they were included as one component of a broader psychomotor profile using an INPP-related framework (Blythe, 2005; Goddard Blythe, 2005). As outlined in the Introduction, the developmental significance of residual reflex activity at ages 5–7 is contested: such activity is common in children without any diagnosis (Gieysztor et al., 2018; Hickey and Feldhacker, 2022; Pecuch et al., 2020), the evidence linking it to motor competence or school outcomes is cross-sectional and based on small samples, and professional bodies and reviewers have cautioned against equating retained reflexes with functional impairment (American Occupational Therapy Association, 2021; Hyatt et al., 2009). The reflex scores in the present study were therefore not treated as diagnostic: in the classification framework, only substantial retention (scores 3–4) contributed to the motor-difficulty category, and residual activity (score 2) contributed only to the at-risk category. Even so, the inclusion of reflex indicators is likely to have increased the proportion of children in the two higher categories and may have contributed to the two-category discrepancies with the teachers’ ratings, because residual reflex activity is not directly observable in everyday classroom behavior. Persistent or partially persistent reflex responses should not be interpreted automatically as evidence of central nervous system immaturity, future learning difficulty, or lack of school readiness. Their relationship with functional motor performance and educational outcomes requires more rigorous investigation–ideally with blinded raters, independent validated motor tests, and longitudinal follow-up–than the present exploratory design can provide.

Several factors may have contributed to discrepancies between the two classification procedures. Teachers observed children across everyday situations, whereas the examiner rated performance during structured tasks; the procedures also used different scoring systems and thresholds. In addition, developmental variability is considerable at ages 5–7, and educator experience may influence the interpretation of observed motor behavior (Honrubia Montesinos et al., 2023; Junaid et al., 2000; Rodríguez-Guerrero et al., 2023). These factors offer plausible explanations for partial rather than complete agreement without implying that either procedure was inherently more accurate.

From a practical perspective, the MABC-2 Checklist may be useful as part of a multi-method screening process because it captures everyday motor behavior in naturalistic settings. However, the present findings do not support its use as a standalone diagnostic instrument or as the sole basis for school-entry decisions. In particular, the pattern of discrepancies suggests that a Checklist result of “no motor difficulty” should not be taken as sufficient grounds to omit further observation when other concerns exist, whereas a Checklist result in the at-risk or motor-difficulty band warrants structured follow-up rather than a conclusion about the child. When concerns are identified, checklist findings should be integrated with standardized motor assessment and broader developmental information.

Further research should prioritize formal Hungarian-language and cultural adaptation of the MABC-2 Checklist, planned inter-rater reliability assessment, use of standardized comparison instruments, larger and more diverse samples, and longitudinal follow-up. These steps are necessary to determine how reliably checklist classifications can be used in Hungarian preschool settings and whether early motor-screening findings have prognostic relevance for later educational functioning.

## 5 Conclusion

In this exploratory sample, teacher-based MABC-2 Checklist classifications showed partial agreement with an examiner-based psychomotor classification. Overall raw agreement was 71.2%, and linearly weighted Cohen’s kappa was 0.503 (95% CI 0.315–0.677), corresponding to moderate agreement, with the lowest category-specific agreement in the at-risk group and a sizeable minority of two-category discrepancies in which teachers did not identify children whom the examiner classified as showing motor difficulty. These findings indicate that the two approaches capture overlapping but not identical aspects of children’s motor functioning.

The results should not be interpreted as formal validation of the MABC-2 Checklist, as evidence of overall school readiness, or as prediction of later academic outcomes. The checklist may have practical value as one component of a broader, multi-method assessment process, but further Hungarian adaptation, reliability research, standardized comparison procedures, larger samples, and longitudinal studies are required before broader standalone educational or clinical use can be recommended.

## 6 Limitations and future directions

A few limitations should be considered when interpreting the present findings.

First, the study used a small non-probability sample drawn from only three urban kindergarten classes in one county. Because no probabilistic sampling strategy was applied and the sample was not representative of rural areas or of the broader socioeconomic diversity of the Hungarian preschool population, the results cannot be generalized beyond this limited context. Future studies should involve larger, multi-site, probabilistic samples that include both urban and rural settings.

Second, the design was cross-sectional. As a result, the study cannot establish predictive validity for later school performance and cannot determine whether the observed psychomotor patterns are associated with later academic or adaptation outcomes. Longitudinal follow-up would be required to assess whether movement-screening results at preschool age relate to school-entry functioning or longer-term educational trajectories.

Third, the examiner-based psychomotor classification was created for the purposes of this exploratory study and combined primitive reflex observations with gross-motor task scores into a composite category. Although this approach was useful descriptively within the present study, it does not constitute a standardized diagnostic reference instrument: the decision criteria in Table 1 were pragmatic working rules rather than validated cut-offs, the procedure has no established inter-rater or test–retest reliability, and the final category involved examiner judgment. The agreement estimates must therefore be read as convergence between two non-standardized procedures rather than as validity coefficients. The component measures assess related but not psychometrically equivalent constructs, and their combination should be interpreted cautiously. In addition, the inclusion of primitive-reflex indicators rests on a framework whose construct validity and educational relevance are contested; the classification would differ if reflex findings were excluded. Future research should examine reflex indicators and gross-motor performance both separately and jointly, using validated instruments where available.

Fourth, rater effects may have influenced both forms of assessment. Teacher checklist ratings depend on everyday observation and may vary with educators’ familiarity with motor development, while examiner-based scoring also involved professional judgment in task administration and interpretation. Because the two teachers of each class produced a single consensus-based Checklist rating per child rather than independent parallel ratings, inter-rater reliability between individual educators could not be estimated in the present study. The teachers received only a brief orientation rather than formal rater training, and the examiner was not blind to the children’s classroom behavior. Future studies should include planned inter-rater reliability analyses, rater training, and multi-rater designs that involve teachers, physical education specialists, and parents.

Fifth, potentially relevant background variables–including gestational age, prematurity, birth weight, perinatal complications, developmental history, socioeconomic background, and habitual physical activity-were not systematically collected. These factors may have affected psychomotor performance and should be included in future studies to allow more nuanced interpretation of observed motor patterns.

Sixth, the MABC-2 Checklist was used without a formal Hungarian-language adaptation process in the present study. The item wording was translated for the purposes of the study without back-translation or psychometric evaluation (see section “2.3 MABC-2 Checklist”), and the translation and cultural appropriateness of item wording, response options, and normative interpretation for Hungarian preschool settings remain to be established. Items referring to activities that are rare in Hungarian kindergarten routines may have increased “not observed” responses and thereby influenced category assignment. Future research should address linguistic and cultural adaptation more explicitly.

Seventh, although linearly weighted Cohen’s kappa was used to quantify agreement between the ordered categories, the modest sample size resulted in a relatively wide 95% confidence interval. The sensitivity, specificity, and predictive values reported here are exploratory and depend on a non-validated comparator, and subgroup analyses by sex and age group were not undertaken because the resulting subgroups (30–43 children) would have been too small to yield meaningful estimates. Larger samples are needed to estimate the strength of agreement more precisely and to examine whether agreement varies across subgroups or settings.

Finally, the score thresholds used in the exploratory procedures–the descriptive threshold of ≥2 in Table 2 and the classification criteria in Table 1–were selected pragmatically within this study and should not be interpreted as formally validated diagnostic cut-offs. The meaning of a score of 2 on the gross-motor tasks may not be directly comparable to a score of 2 on the primitive-reflex assessment, and the psychometric equivalence of these scales has not been demonstrated. Future research should examine the sensitivity and specificity of alternative thresholding approaches, ideally against an independent, standardized motor test.

Future research directions include: (a) larger and more diverse samples with clearer sampling frames; (b) formal Hungarian adaptation and validation of the MABC-2 Checklist, including inter-rater reliability studies across different evaluator types; (c) longitudinal designs to examine the predictive value of preschool movement-screening results for later school functioning; (d) separate and combined examination of reflex and gross-motor indicators using standardized instruments; and (e) collection of perinatal, developmental, and socioeconomic variables to permit more comprehensive risk-adjusted analyses.

## Data Availability

The raw data supporting the conclusions of this article will be made available by the authors, without undue reservation.
